# Kinetics of MM1.S Multiple Myeloma Cells in a 3D Polymer Particle Culture System with Bone Marrow Stromal Cells and Bortezomib

**DOI:** 10.3390/ph19010122

**Published:** 2026-01-10

**Authors:** Shin Aizawa, Miyuki Yuda, Shuichi Hirai, Isao Tsuboi, Takashi Koike, Yoshihiro Hatta, Katsuhiro Miura, Masahiro Yasuda

**Affiliations:** 1 Division of Anatomical Science, Department of Functional Morphology, Nihon University School of Medicine, Tokyo 173-8610, Japan; yuda.miyuki@nihon-u.ac.jp (M.Y.); i-tsuboi@dd.em-net.ne.jp (I.T.); 2 Division of Hematology and Oncology, Department of Internal Medicine, Nihon University School of Medicine, Tokyo 173-8610, Japan; 3 Department of Chemical Engineering, Osaka Metropolitan University, Osaka 599-8531, Japan; m-yasuda@omu.ac.jp

**Keywords:** 3D culture, bone marrow stromal cell, polymer particles, multiple myeloma, bortezomib, apoptosis

## Abstract

**Background**: Three-dimensional (3D) culture systems use polymer particles with a bone marrow stroma cell feeder layer to reproduce a biostructural hematopoiesis state more effectively than in conventional two-dimensional (2D) culture methods. The 3D culture maintains normal hematopoiesis, resulting in prolongation of hematopoietic stem cell proliferation and differentiation, while the bone marrow stromal cells in the culture alter the growth of leukemic cells and protect them from anticancer agents. However, the effect of stromal cells on hematopoietic stem cell proliferation and differentiation and neoplastic cells, including leukemia, in 3D culture is still a point of contention. **Methods**: We assessed the mechanism of two different bone-marrow-derived stromal cells (i.e., MS-5 and Tst-4) with different characteristics by using a feeder layer in the 3D culture to compare their supportive action on leukemic cells, focusing on the role of 3D cultures constructed with bone marrow stromal cells in leukemic cell growth. Multiple myeloma cells are strongly related to stromal cells in their proliferation; hence, cloned MM1.S cells derived from multiple myeloma were cocultured in 3D, and their cell growth was examined. We also examined the effect of the antineoplastic agent bortezomib, a proteasome inhibitor, in the 3D culture system with a different stromal cell feeder. **Results and Conclusions**: When MM1.S myeloma cells were cultured with MS-5 stroma in 3D conditions, cell growth was found to be slow compared with that in 2D culture, as well as with those in both the 2D and 3D cocultures with Tst-4 stroma. Additionally, the MS-5 cells in the 3D culture protected the MM1.S cells from the cytocidal effect of the bortezomib treatment. Different MM1.S cell kinetics were observed depending on the stromal cells used, suggesting their inherent and complicated characteristics.

## 1. Introduction

Hematopoietic stem cells (HSCs) possess the capacity for self-renewal and the ability to produce progenitors committed to differentiation into diverse blood cell lineages, including erythrocytes, leukocytes, lymphocytes, and platelets; their proliferation and differentiation within hematopoietic tissue are regulated by the bone marrow microenvironment [1,2,3]. Stromal cells are distinct from hematopoietic cells and are an essential component of this microenvironment, necessary for the long-term maintenance of HSCs in vitro [4,5,6]. Several studies have shown that bone marrow stromal cells regulate HSC proliferation and differentiation by producing growth factors, extracellular matrix, and physical cell–cell interactions through adhesion molecules and gap-junction-mediated cell communication [1,6,7,8,9,10,11,12]. Furthermore, stromal cells’ physiological function means that they require an anatomically appropriate three-dimensional (3D) orientation to determine their fate, which is linked to a normal and/or dysfunctional hematopoietic system [10,13,14,15,16]. The effect of marrow stromal cells on leukemic cell proliferation, as well as that of normal hematopoietic cells, has been reported previously, and multiple myeloma cell and stromal cell growth are also strongly related [17]. In addition, stromal cells likely protect leukemic cells from the effects of cytotoxic anticancer agents [18,19]. However, the regulation mechanism of stromal cells on HSC proliferation and differentiation is not well understood. Furthermore, the growth-regulating and protective actions of stromal cells toward leukemic cells have not been elucidated at all.

We recently developed novel polymer particles with grafted epoxy polymer chains to support cell immobilization in a new 3D cell cultivation system [20,21,22]. The base polymer particles were synthesized by the suspension polymerization of an acrylic monomer and 2,2′-azobis[N-(2-propenyl)-2-methylpropionamide], and the epoxy polymer chain was extended from the particle surface through graft polymerization. An advantage of these particles is that their size, length, number of chains, and base polymer composition can be easily manipulated. Additionally, stromal cells are easy to bind and grow on these particles’ surfaces, building strong bridges between particles and forming a 3D environment. Normal hematopoiesis is maintained in our 3D culture system, resulting in the prolongation of the hematopoietic stem cell proliferation and differentiation [20], and the bone marrow stromal cells in the culture alter the growth of leukemic cells and protect them from the cytocidal action of anticancer agents [23,24].

In this study, two different 3D culture systems were constructed using two different stromal cells, namely MS-5 and Tst-4, to examine their specific functions: Tst-4 cells promote B-cell differentiation [25,26], and MS-5 cells support murine and human HSC proliferation and differentiation and regulate leukemic cell growth in vitro [20,27,28]. MM1.S cells derived from a patient with multiple myeloma were used as the target hematopoietic cells [29], as the MM1.S cell line is widely used in multiple myeloma research, including drug action and resistance studies. In addition, two bortezomib-resistant cell lines (MM1.S/BTZ and NCI-H929/BTZ) were generated and used to analyze the cytocidal action of bortezomib [30]. The MM1.S cells were cocultured with MS-5 or Tst-4 stromal cells in 3D or two-dimensional (2D) culture to assess their action on MM1.S cell proliferation, and the mouse-origin MS-5 and Tst-4 cells and human-origin MM1.S cells were identified using distinct antibodies as a human- and murine-specific cell surface markers from the pool of 3D and 2D cocultured cells [23,24].

Bortezomib, a novel proteasome inhibitor, has been developed as an anticancer agent and widely used against multiple myeloma [31,32]. However, the drug’s curative effect is currently limited to in vivo and in vitro settings [33,34,35,36], while the reason for treatment resistance in multiple myeloma patients remains unclear. We examined the effect of bortezomib on MM1.S cells using a 3D coculture approach with stromal cells, primarily aiming to unravel the effect of MS-5 and Tst-4 presence on the growth of MM1.S cells with or without bortezomib treatment. Secondly, we also aimed to determine whether we could reproduce an in vivo drug effect by employing a 3D in vitro coculture system.

## 2. Results

### 2.1. Growth Patterns of MM1.S Cells Cocultured with MS-5 and Tst-4 in 2D and 3D Culture Systems

**Figure 1:**
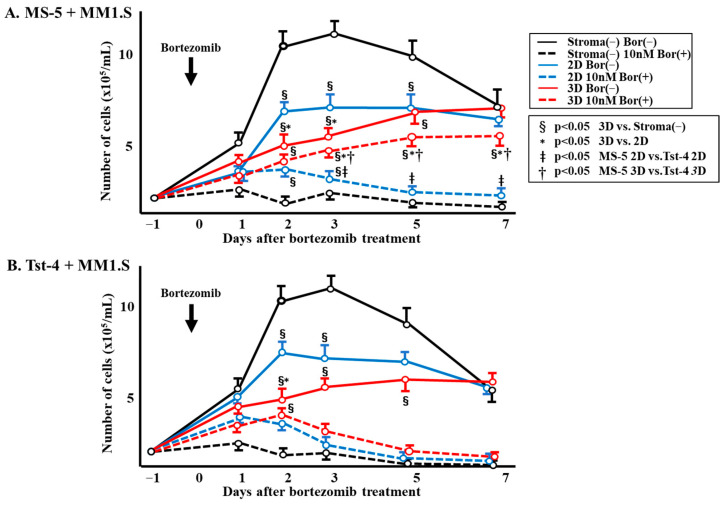
Number of viable cells after treatment with or without bortezomib. (**A**) MM1.S cells (1 × 10^5^/mL) were cultured with MS-5 stromal cells in a 2D or 3D culture system. Bortezomib (10 nM/mL) was introduced, cells were harvested daily for 7 days, and the number of viable cells was counted by trypan blue dye exclusion. (**B**) MM1.S cells (1 × 10^5^/mL) were cultured with Tst-4 stromal cells in a 2D or 3D culture system. Bortezomib (10 nM/mL) was introduced, cells were harvested daily for 7 days, and the number of viable cells was counted by trypan blue dye exclusion. Two different wells were prepared for each point, and experiments were performed in triplicate. The error bars show the mean ± SD. §: Significant difference (*p* < 0.05) between 3D and stroma-cell-free culture. *: Significant difference (*p* < 0.05) between 3D and 2D culture. ǂ: Significant difference (*p* < 0.05) between MS-5 2D and Tst-4 2D. †: Significant difference (*p* < 0.05) between MS-5 3D and Tst-4 3D.

Approximately 1 × 10^5^/mL of MM1.S cells were cocultured with either MS-5 or Tst-4 stromal cells in 2D and 3D cultures, and Figure 1 shows clear differences between the culture systems in the numbers of viable MM1.S cells. Due to the cell line’s natural doubling time of approximately 72 h [29], the MM1.S cells cultured without stromal cells initially demonstrated high proliferation; however, the cells reached a plateau by day 2 (4 days after cultivation), followed by a gradual decrease due to the limiting nutritional environment (black line). Upon coculturing with MS-5 cells, the MM1.S showed a slower growth rate (Figure 1A, solid blue (2D) and red lines (3D)), especially in the 3D culture, where viable cells kept proliferating until day 7 (solid red line); a similar growth pattern of MM1.S cells was observed when cocultured with Tst-4 (Figure 1B). The MM1.S cell proliferation in the 2D culture was faster than that observed in the 3D culture (solid blue line).

### 2.2. Growth Patterns of MM1.S Cells Cocultured with MS-5 and Tst-4 Cells in 2D and 3D Culture Systems Along with Bortezomib Treatment

**Figure 2:**
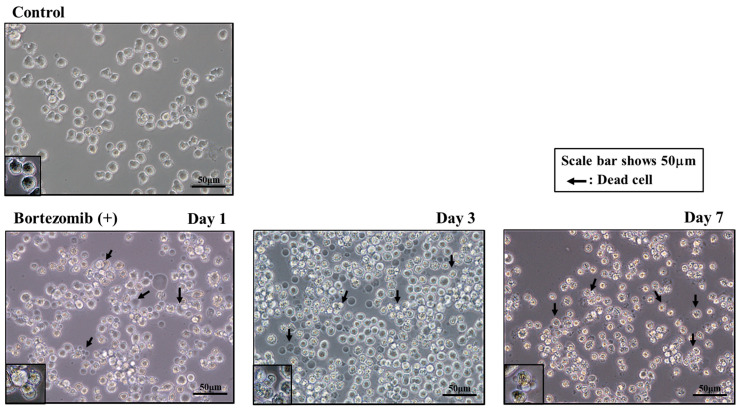
Phase contrast images of MM1.S cells in stromal-cell-free culture after treatment with 10 nM/mL of bortezomib after 1, 3, and 7 days. Scale bars show 50 μm, and arrows show the smaller and irregularly shaped MM1.S cells (dead cells) induced by bortezomib treatment.

**Figure 3:**
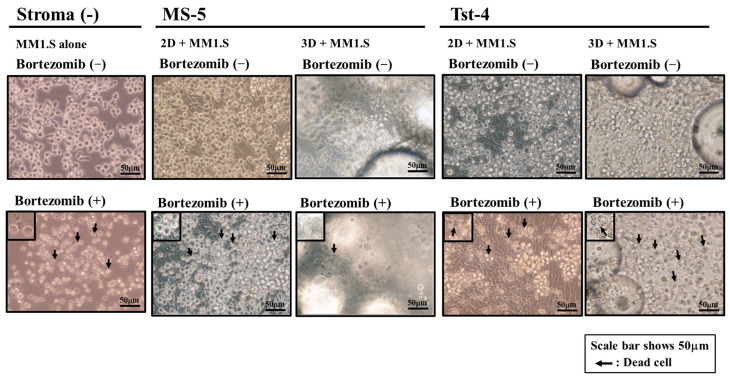
Phase contrast images of MM1.S cells 3 days after bortezomib treatment in 2D or 3D cultures constructed with MS-5 or Tst-4 stromal cells. Scale bars show 50 μm and arrows show the smaller and irregularly shaped MM1.S (dead cells) induced by bortezomib treatment.

This experiment allowed us to examine the influence of the anticancer agent bortezomib, a proteasome inhibitor, on MM1.S growth. Kim et al. reported that bortezomib inhibit the proliferation of MS-5 stromal cells [37]. Thus, to evaluate the drug’s cytotoxicity, it was introduced to 2D and 3D cultures constructed with MS-5 and Tst-4 cells. Trypan blue dye exclusion assays showed that 85.7% and 79.5% of MS-5 and 85.7% and 80.4% of Tst-4 in the 2D and 3D cultures, respectively, survived after 10 nM bortezomib treatment for 5 days. Next, 1 × 10^5^/mL of the MM1.S cells were cocultured with MS-5 or Tst-4 stromal cells in the 2D and 3D culture systems, and the following day (day 0), when the MM1.S cells adhered to the stromal cells, 10 nM of bortezomib was introduced into each culture. The MM1.S cells were collected and assayed, and Figure 2 shows the phase contrast images of the cells in the stromal-cell-free culture with bortezomib after 1, 3, and 7 days. The MM1.S cells appeared irregular in shape with a slight reduction in their size, and the trypan blue dye exclusion assay revealed that most of the deformed cells were dead. Figure 3 depicts the phase contrast images of the MM1.S cells cocultured with MS-5 and Tst-4 stromal cells 3 days after bortezomib treatment in the 2D and 3D culture systems. The MM1.S cells remained adherent to the MS-5 stromal cells in the 3D culture and maintained a rounded shape, while in contrast, those in the 2D culture with MS-5 and those in both the 2D and 3D culture constructed with Tst-4 cells showed morphological changes, appearing small and irregularly shaped, as seen in the stromal-cell-free culture with bortezomib treatment.

Figure 1 displays the alterations in the viable MM1.S cell numbers after bortezomib treatment. The MM1.S cell numbers in the stromal-cell-free culture were rapidly reduced after bortezomib treatment (dotted black line). The number of viable cells in the 3D coculture MS-5 was significantly higher (*p* < 0.05) than without stromal cells and in the 2D culture with MS-5 cells, and it gradually increased until day 7 post-treatment (dotted red line). Contrarily, the number of viable MM1.S cells cocultured in 3D with Tst-4 stromal cells significantly reduced (*p* < 0.05) within 3 days after bortezomib treatment (Figure 1B). In addition, the number of viable MM1.S cells in the culture with MS-5 stroma were significantly higher than those achieved with Tst-4 stroma, both in 2D and 3D.

**Figure 4:**
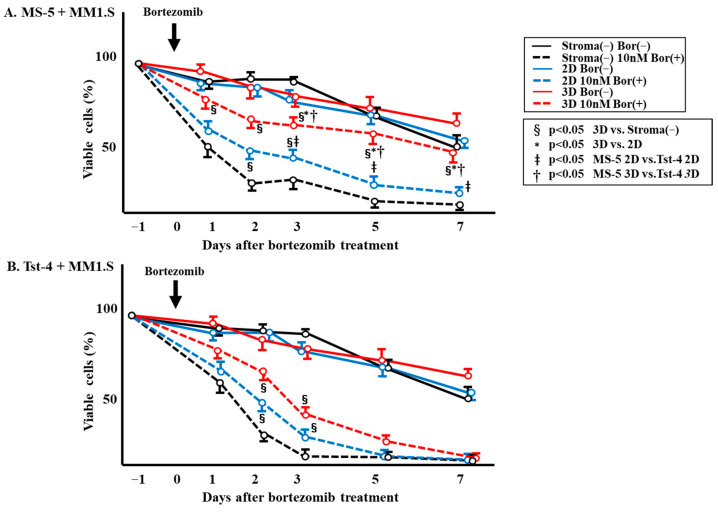
The % of viable cells after treatment with or without bortezomib in 2D or 3D cultures constructed with MS-5 or Tst-4 stromal cells. (**A**) MM1.S cells (1 × 10^5^/mL) were cultured with MS-5 stromal cells in 2D or 3D. Bortezomib (10 nM/mL) was introduced, cells were harvested daily for 7 days, and the % of viable cells was calculated: number of viable cells/total number of cells × 100 (**B**). MM1.S cells (1 × 10^5^/mL) were cultured with Tst-4 stromal cells in 2D or 3D. Bortezomib (10 nM/mL) was introduced, cells were harvested daily for 7 days, and the % of viable cells was calculated. Two different wells were prepared for each point, and experiments were performed in triplicate. The error bars show the mean ± SD. §: Significant difference (*p* < 0.05) between 3D and stroma-cell-free culture. *: Significant difference (*p* < 0.05) between 3D and 2D culture. ǂ: Significant difference (*p* < 0.05) between MS-5 2D and Tst-4 2D. †: Significant difference (*p* < 0.05) between MS-5 3D and Tst-4 3D.

Figure 4 shows the percentage of viable MM1.S cells after bortezomib treatment, which was found to be reduced in the culture without stroma and in the 2D and 3D cocultures with Tst-4 cells (Figure 4B). In contrast, the percentage of viable MM1.S cells in the 3D coculture with MS-5 stromal cells was significantly higher (*p* < 0.05) compared to that observed in culture without stromal cells and in the 2D and 3D cocultures with Tst-4 (Figure 4A). More than 40% of cells were viable in the 3D coculture with MS-5, compared to only 10.3% in 2D coculture with MS-5 and 2.6% in 3D coculture with Tst-4 cells. It was also found that the % of viable MM1.S cells in the culture with MS-5 stroma was significantly higher than for cells cocultured with Tst-4 in both 2D and 3D.

### 2.3. Expression of the Apoptotic Marker 7A6 Antigen on the MM1.S Cells After Bortezomib Treatment

**Figure 5:**
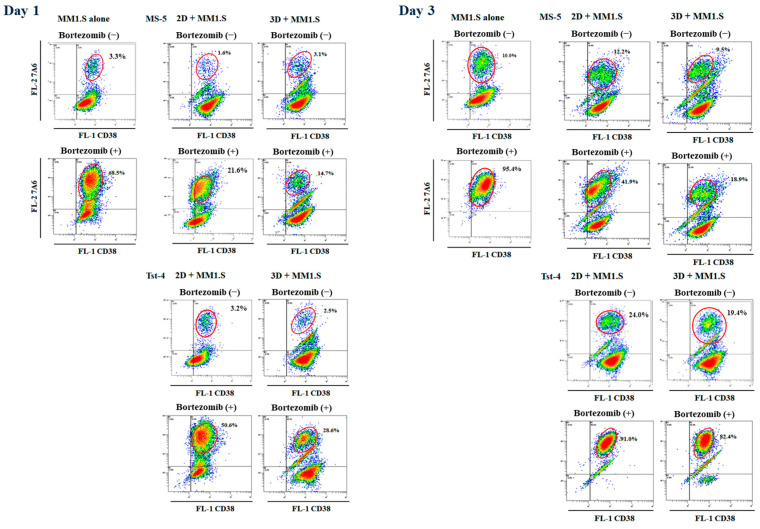
Typical dot plot histograms of two-color flow cytometry of the MM1.S cells determined by CD38 (FL-1) and APO2.7 (FL-2) 1 and 3 days after bortezomib treatment. Bortezomib was added to the culture 24 h after cell inoculation.

**Figure 6:**
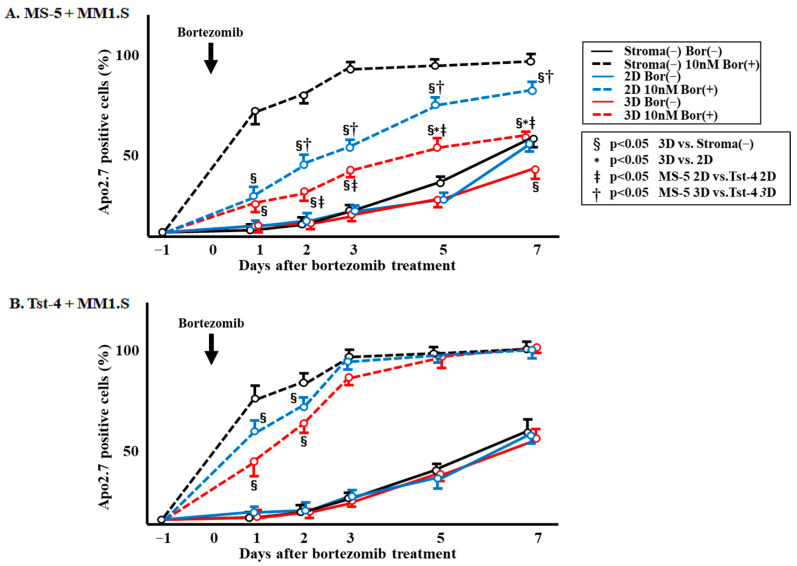
The % of 7A6-positive cells among CD38-positive cells after treatment with and without 10 nM/mL bortezomib. (**A**) MS-5 + MM1.S. (**B**) Tst-4 + MM1.S. Two different wells were prepared for each point, and experiments were performed in triplicate. The error bars show the mean ± SD. §: Significant difference (*p* < 0.05) between 3D and stroma-cell-free culture. *: Significant difference (*p* < 0.05) between 3D and 2D culture. ǂ: Significant difference (*p* < 0.05) between MS-5 2D and Tst-4 2D. †: Significant difference (*p* < 0.05) between MS-5 3D and Tst-4 3D.

We investigated the mechanisms underlying the reductions in MM1.S cell numbers in response to bortezomib treatment by examining 7A6 (apoptosis antigen) expression. A cell aliquot was collected from each culture 1, 2, 3, 5, and 7 days after treatment and double-stained with anti-CD38 and anti-7A6 antibodies (APO2.7) (Figure 5 and Figure 6). The human leukemic MM1.S cells were positive for CD38 [29,36], whereas the murine MS-5 and Tst-4 cells were negative, making them easy to distinguish. Figure 5 shows a typical dot plot histogram of the two-color cytometry of the MM1.S cells determined by CD38 (FL-1) and APO2.7 (FL-2) 1 and 3 days after bortezomib treatment, and Figure 6 summarizes the results. Significantly (*p* < 0.05) higher numbers of APO2.7-positive cells were observed in the culture without stromal cells and in the 2D and 3D cultures with Tst-4, suggesting that apoptosis was induced by bortezomib under these conditions and that bortezomib induces apoptosis in MM1.S myeloma-derived cells. In addition, there were significantly more APO2.7-positive cells in the Tst-4 stroma culture compared with the MS-5 culture. Thus, MS-5 cells in 3D culture systems may protect MM1.S cells from the apoptosis induced by bortezomib treatment.

## 3. Discussion

Hematopoiesis occurs in the bone marrow, and distinctive stromal cells associate HSC proliferation and differentiation [1,2,3]. Schofield proposed the concept of a “niche” as a specialized microenvironment housing HSCs [38], and several in vitro experiments coculturing hematopoietic cells with stromal cells have demonstrated that both stromal cell function and niche anatomy regulate stem cell quiescence, self-renewability, and differentiation [13,14,15,16]. Abnormal clones originating from hematopoietic cells, including leukemic clones, arise within the niche, indicating that the stromal cells regulate proliferation of both normal hematopoietic cells and these abnormal clones [18,39]. However, not all stromal cells derived from hematopoietic tissue support hematopoiesis and leukemogenesis. In some stromal cells, inhibitory action on hematopoietic growth as well as abnormally cloned cells have been reported [40].

Herein, we developed a novel 3D culture system using polymer particles grafted with epoxy polymer chains for cell immobilization. In this system, HSCs and cloned leukemic cells grow better than in traditional 2D culture systems [20,22]. In our 3D cultures, stromal cells regulate HSC and cloned leukemic cell growth by regulating the cell cycle [23,24]. Two types of stromal cells, namely MS-5 and Tst-4, were cocultured with multiple-myeloma-derived cloned MM1.S cells to compare their specific functions in 3D culture. Coculturing the MM1.S cells with MS-5 and Tst-4 cells in the 2D culture showed a comparable proliferation pattern (Figure 1 and Figure 4); however, in 3D coculture, MM1.S cells grew slower with MS-5 cells compared to with Tst-4 cells. Interestingly, the relaxed growth pattern of the MM1.S cells when 3D cocultured with MS-5 was comparable to that observed in 2D coculturing. Altogether, these findings indicate that MM1.S cell growth varies according to stromal cell type, along with culture system dimensions.

Bortezomib, a proteasome inhibitor, was developed to effectively treat multiple myeloma [31,34]. Various studies have reported the drug’s cytotoxic effects, such as inducing canonical nuclear factor-kappa B (NF-κB) activation in multiple myeloma cells [32,41], inducing apoptosis by oxidative and ER stress [33,42,43], and overcoming cell-adhesion-mediated drug resistance by downregulating VLA-4 expression [44]. Weeks et al. reported that VLA-4-mediated adhesion to bone marrow stromal cells confers chemoresistance to adherent lymphoma cells [45], providing evidence that MS-5 cells protected adhered lymphoma cells from vincristine treatment. Zhou et al. showed that bortezomib suppresses the leukemic cells’ self-renewability by promoting NF-κB-dependent CDK6 inhibition [46]. Previously, we reported that MS-5 stromal cells in a 3D coculture influenced the proliferation and differentiation of hematopoietic cells by regulating their cell cycle, wherein more than 50% of the cells failed to enter the S phase [23]. Indeed, a reduced apoptotic change (7A6 antigen expression) was observed in MM1.S cocultured with MS-5 cells in 3D system. The protective effect of MS-5, but not Tst-4, against the cytocidal treatment of bortezomib may be related to the regulation of the MM1.S cell cycle period by MS-5 cells in the 3D microenvironment. Interestingly, we found that bortezomib induces interleukin(IL)-6 production, which supports myeloma cell proliferation, from bone marrow stromal cells [17]. It is necessary to examine cytokine production in MS-5 and Tst-4 stromal cells after bortezomib treatment in both 2D and 3D culture systems, and understanding the intricacies of this cellular cross-talk is imperative and needs further investigation. Our results revealed that the stromal MS-5 and Tst-4 cells in the 2D coculture partially protected the MM1.S cells from bortezomib-induced apoptosis, but greater protection was observed only in the 3D system with MS-5 cells. Thus, the anatomical niche constructed with MS-5 cells, but not that with Tst-4 stroma cells, is important for regulating target hematopoietic cells. The possibility of a change in the characteristics and functions of MS-5 cells under different culture conditions (2D or 3D) remains to be investigated. We showed that the MS-5 stroma in the 3D system protected MM1.S cells from bortezomib-induced apoptosis better than the MS-5 cells in the 2D culture, as well as the Tst-4 cells in 2D and 3D culture systems. It is well known that apoptosis is caused by various mechanisms, but unfortunately we were only able to examine the expression of the 7A6 antigen on MM1.S cells after bortezomib treatment. Various mechanisms have been proposed for the protective action of MS-5 cells on MM1.S cells in a 3D system, e.g., direct cell-to-cell interaction through adhesion molecules, paracrine factors produced by MS-5 cells, or signal pathway regulation. It is likely that additional analysis of apoptotic cells by the TUNEL (Terminal deoxynucleotidyl transferase dUTP nick end labeling) method, DNA Laddering detection, and measurement of apoptosis-related enzyme (caspase) activity, mitochondrial cytochrome c release, and stromal cell cytokine production will provide important information on cell-to-cell interaction mechanisms in 3D culture systems. However, this is the first report to demonstrate that stromal cells’ potential in mediating the resistance of multiple myeloma cells against bortezomib is dependent on the culture system (2D or 3D) and stromal cell type (MS-5 or Tst-4). In this study, mouse-origin MS-5 and Tst-4 cells and human-origin MM1.S cells were used, and the harvested MM1.S cells were easy to distinguish from the pool of 3D and 2D cocultured cells using distinct antibodies as human- and murine-specific cell surface markers. On the other hand, several reports have shown the utility of 3D microenvironments constructed with human stroma for studying tumor–stroma interactions [47,48], and a 3D human stroma culture system using polymer particles should be considered in a future study. In addition, a novel 3D culture system using fresh myeloma cell samples from myeloma patients may prove to be a useful tool for studying the mechanism of myeloma–stroma cell interaction.

Further studies are necessary to investigate the mechanisms that regulate the hematopoietic or leukemic cell homing to the stromal niche, including multiple myeloma cells, as well as the role of stroma in maintaining these cells in a stable condition. Comparative studies using both 2D and 3D culture systems with various stromal cells may aid in unraveling these questions.

## 4. Materials and Methods

### 4.1. Materials

2,2′-Azobis (isobutyronitrile), 2,2′-azobis[N-(2-propenyl)-2-methylpropionamide], dipotassium hydrogen phosphate, glutaraldehyde aqueous solution (25% *w*/*v*), glycidyl methacrylate, hydrochloric acid, methacrylic acid, methanol, methyl methacrylate, paraformaldehyde aqueous solution (36% *w*/*v*), saccharose, sodium dihydrogen phosphate, and trypan blue were purchased from Wako Pure Chemical Industries, Ltd. (Osaka, Japan). Cresol red, poly (vinylpyrolidone) K-90, sodium hydroxide, and toluene were purchased from Nacalai Tesque (Kyoto, Japan). Pentaerythritol triacrylate was purchased from Sigma-Aldrich Co. (St. Louis, MO, USA) [24].

Bortezomib was purchased from Selleck Biotechnology Co. (Yokohama, Japan), dissolved in dimethyl sulfoxide (DMSO; Sigma-Aldrich Co.) to prepare a 5 μM solution, and then mixed with Iscove’s modified Dulbecco’s medium (IMDM, Invitrogen Corp., Carlsbad, CA, USA) for use. A previous study showed that the half-maximal effective concentration (EC50) of bortezomib for U266 myeloma cells was 2.45 nM [36]. In the present study, bortezomib was used at a 10 nM concentration.

### 4.2. Preparation of Cell Support with Grafted Polymer Chains

Polymer particles with grafted epoxy polymer chains were prepared as previously described [21,24]. For graft polymerization, glycidyl methacrylate and methacrylic acid were used at a ratio of 4:1 (*w*/*w*), and the resulting particles were washed in a funnel with a 40× volume of distilled water and methanol. These polymer particles are herein referred to as G-02 polymer particles.

Particle diameter distribution was measured using a Microtrac FRA laser particle diameter analyzer (Microtrac, Inc. (Montgomery, PA, USA)), and 100–200 μm particles were used in this study. The amount of epoxy in the polymer particles was measured using the hydrochloric acid–dioxane method. Particles with more than 1.03 × 10^−3^ μmol/g-particle of the epoxy group were selected [20,21,24].

### 4.3. Culturing of MS-5 and Tst-4 Stromal Cells and MM1.S Cells

The murine stromal cell lines, MS-5 and Tst-4, were cultured using 7 mL of IMDM supplemented with 10% fetal calf serum (FCS; Hyclone (Logan, UT, USA)), penicillin (50 U/mL; Sigma-Aldrich Co.), and streptomycin (100 μg/mL; Sigma-Aldrich Co.) in 25-cm^2^ flasks (Falcon 3013; Corning (One Riverfront Plaza, NY, USA)). The cells were maintained in a humidified incubator at 37 °C with 5% CO_2_, and subcultured at a split ratio of 1:4 every 7 days using 0.25% trypsin plus 0.02% EDTA in phosphate-buffered saline (PBS) [24].

Human myeloma-derived MM1.S cells [29] were cultured using 7 mL of IMDM supplemented with 10% FCS, penicillin (50 U/mL), and streptomycin (100 μg/mL) in 25-cm^2^ flasks. The cells were maintained in a humidified incubator at 37 °C and 5% CO_2_, and then subcultured at a split ratio of 1:10 every 5 days. For all experiments, the cells were used in their logarithmic growth phase.

### 4.4. Coculture System

The MS-5 and Tst-4 cells (5–10 × 10^5^) were added to 5 mL of IMDM supplemented with 10% FCS in the presence of 1–5 × 10^4^ G-02 polymer particles in a 14 mL round-bottomed polypropylene tube (Falcon 352059). The mixture was incubated in a humidified incubator at 37 °C and 5% CO_2_ for 24 h, transferred to 35 mm plastic dishes (Falcon 353046), and incubated. Once the cells were immobilized on the particle surface, they proliferated and formed bridges between the particles. The supernatant was replaced with fresh growth medium every 7 days, and after 2–3 weeks, when the MS-5 and Tst-4 stromal cells had formed a 3D layer in the culture dish, MM1.S cells (1 × 10^5^/mL) suspended in 5 mL of growth medium were layered over the stromal cells [24]. Control cultures included MS-5 and Tst-4 cells grown in dishes without G-02 particles, and MM1.S cells were also inoculated over stromal cells in traditional 2D cultures.

### 4.5. Bortezomib Treatment

The MM1.S cells were inoculated into the culture one day before bortezomib treatment at 1 × 10^5^/mL cell density, and the cultured cells were collected at various intervals for analysis. The adherent MM1.S cells could be dislodged from the MS-5 and Tst-4 stromal cells in both the 2D and 3D cultures without trypsin treatment and were harvested by repeated pipetting. The harvested cells were counted using a hemocytometer, and viable cells were identified using the trypan blue dye exclusion method. The MM1.S and stromal cells could easily be distinguished by size and shape under microscopic observation. A cell aliquot was assayed for CD38 and APO2.7 antigen expressions by flow cytometry, with two different wells prepared for each point and experiments performed in triplicate.

### 4.6. Flow Cytometry

The harvested cells were washed with PBS containing 2% FCS and passed through a 35 μm filter (Cell Strainer; Falcon 352235) to remove the polymer particles and aggregated cells, most of which were MS-5 and Tst-4. Cells (2 × 10^5^) were suspended in 0.5 mL PBS containing 2% FCS and 0.02% NaN_3_ and incubated with fluorescein isothiocynate (FITC)-conjugated anti-human CD38 (clone HIT2, Becton Dickinson Labware, Franklin Lake, NJ, USA) for 30 min at 4 °C for the detection of MM1.S cells, as these cells express the CD38 antigen, whereas MS-5 and Tst-4 do not. Cells were washed with PBS twice, resuspended in 100 µL of PBS, and then, to detect intracellular 7A6 antigen, an Intracellular Fixation and Permeabilization Buffer Set (catalog number: 88-8824; Thermo Fisher Scientific, Rockford, IL, USA) was used according to the manufacturer’s instructions [49]. The washed cells were fixed by adding 100 µL of Fixation Buffer, and incubated for 30 min at room temperature in the dark. Then, 2 mL of Permeabilization Buffer was added, and the cells were centrifuged at 500× *g* for 5 min at room temperature. After the cells were resuspended in 100 µL of Permeabilization Buffer, PE-conjugated APO2.7 monoclonal antibody (Clone CXNFT, catalog number 25-5920-82; Thermo Fisher Scientific) was added, and the cells were incubated for 30 min at room temperature in the dark. Subsequently, 2 mL of Permeabilization Buffer was added, and the cells were centrifuged at 500× *g* for 5 min at room temperature. The supernatant was then discarded, and the cells were resuspended in 2 mL of Permeabilization Buffer and centrifuged at 500× *g* for 5 min at room temperature. After the cells were resuspended in PBS, they were analyzed by flow cytometry (Cytomics FC500; Beckman Coulter, Brea, CA, USA) for the direct detection of CD38 and 7A6 antigens.

BD Pharmingen™ PE Mouse IgG2a, κ Isotype Control (Becton Dickinson, catalog number 559315), and BD Pharmingen™ FITC Mouse IgG2, κ Isotype Control (Becton Dickinson, catalog number 555593), were used for negative control.

The cells were washed thrice with PBS and assessed by flow cytometry on a Cytomix FC500 (Beckman Coulter, Brea, CA, USA).

Two different wells were prepared for each point, and experiments were performed in triplicate.

### 4.7. Statistical Analysis

The results are expressed as the mean ± SD of triplicate experiments. Differences between the means were determined using two-way analysis of variance (ANOVA), and *p* ≤ 0.05 was considered significant. The results are shown as below:

§: Significant difference (*p* < 0.05) between 3D and stroma-cell-free culture.

*: Significant difference (*p* < 0.05) between 3D and 2D culture.

ǂ: Significant difference (*p* < 0.05) between MS-5 2D and Tst-4 2D.

†: Significant difference (*p* < 0.05) between MS-5 3D and Tst-4 3D.

## 5. Conclusions

In our 3D coculture system, MS-5 stromal cells, but not Tst-4, were found to regulate multiple myeloma cell proliferation when compared to 2D coculturing. In the 3D coculture with MS-5 cells, most MM1.S cells were maintained in a stabilized state. Moreover, the cytocidal activity of bortezomib was reduced compared with its action against the cells grown in 2D culture. Although further studies are required to investigate the mechanisms underlying the cell-cycle-regulatory activity of MS-5 cells in 3D culture, the results indicate that this system could serve as a valuable tool for investigating stromal–leukemic interactions, including those involving multiple myeloma cells, in vitro.

## Data Availability

The original contributions presented in this study are included in the article. Further inquiries can be directed to the corresponding author.
